# Immunogenetics of glioblastoma: the future of personalized patient management

**DOI:** 10.1038/s41698-018-0070-1

**Published:** 2018-12-04

**Authors:** Malak Abedalthagafi, Duna Barakeh, Kara M. Foshay

**Affiliations:** 1 Genomics Research Department, Saudi Human Genome Project, King Fahad Medical City and King Abdulaziz City for Science and Technology, Riyadh, Saudi Arabia; 2 Department of Pathology, Brigham and Women’s Hospital, Harvard Medical School, Boston, MA USA; 3 Inova Neuroscience and Spine Institute, Inova Health Systems, Falls Church, VA USA

## Abstract

The prognosis of glioblastoma has changed little over the past two decades, with only minor improvements in length of overall survival through the addition of temozolomide (temodal) to standard of care and the recommended use of alternating electric field therapy (optune) to newly diagnosed patients. In an effort to define novel therapeutic targets across molecularly heterogeneous disease subgroups, researchers have begun to uncover the complex interplay between epigenetics, cell signaling, metabolism, and the immunosuppressive tumor microenvironment. Indeed, IDH mutations are now recognized as a defining differential factor not only influencing global hypermethylation and patient prognosis but also degree of immune infiltration within individual tumors. Likewise, next-generation sequencing has defined subgroup-specific transcriptional profiles that correlate with different mechanisms of immune evasion, including increased PD-L1 and CTLA-4 among mesenchymal tumors. Interestingly, sequencing of the T cell repertoire from numerous patient samples suggests that the correlation between mutational burden and enrichment of tumor-specific peptides may be less convincing than originally suspected. While this raises questions over the efficacy of dendritic cell or tumor-lysate vaccines and CAR-T therapies, these avenues continue to be explored. In addition to these active immunotherapies, inhibitors of molecular hubs with wide reaching effects, including STAT3, IDO, and TGF-β, are now in early-phase clinical trials. With the potential to block intrinsic biological properties of tumor growth and invasion while bolstering the immunogenic profile of the tumor microenvironment, these new targets represent a new direction for GBM therapies. In this review, we show the advances in molecular profiling and immunophenotyping of GBM, which may lead to the development of new personalized therapeutic strategies.

## Introduction

Glioblastoma (GBM) is the most common primary malignancy of the central nervous system (CNS) in adults, with a median survival of 12–15 months despite multi-modality treatments.^1^ The aggressive nature of GBMs and their relative therapeutic resistance reflect an insidious invasiveness, marked genetic heterogeneity, and relative seclusion and resistance to innate immunoediting strategies.

The diagnosis of glioma, and most primary CNS tumors, is made on the subjective basis of histopathology analysis.^2^ While the presence or absence of necrosis, microvascular proliferation, and other anaplastic features are used to assign tumor grade according to World Health Organization (WHO) guidelines, there is no evidence to support a direct relationship between observations of tumor morphology and in vivo responses to therapy.^3^ Thus major efforts in next-generation sequencing (NGS) have been employed to investigate inter-patient heterogeneity and drivers of tumorigenesis that represent novel therapeutic targets.^4,5^ This in-depth molecular characterization revealed the existence of four to six distinct molecular GBM subtypes classified by canonical genetic and epigenetic changes.^6–8^ This led the WHO publishing new guidelines for the molecular staging and diagnosis of glioma.^9^

New pathological diagnostic approaches driven by these advancements include analysis of isocitrate dehydrogenase (IDH) mutation status (either by immunohistochemistry or sequencing) and *O*^6^-methylguanine-DNA methyltransferase (MGMT) promoter methylation, which are now considered the standard of care. However, quantitative analysis of copy number, mutation status, promoter methylation, and deletions may also be informative for other key genes implicated in the pathophysiology of different GBM subtypes, including epidermal growth factor receptor (EGFR), ATRX, cyclin-dependent kinase 4 (CDK4), CDKN2A/B, and Tert. In addition, longitudinal studies suggest that genetic diversification occurs as tumors evolve and recur, and thus methods to reanalyze or track molecular changes may be necessary. Interestingly, emerging NGS analyses also suggests that inflammatory and immune activation/suppression pathways may vary within tumors of different molecular GBM subtypes. These data, along with the success of immunotherapies in other cancer types, have driven a rapid expansion into GBM immunology.

The role of the immune system in glioma pathophysiology was historically underappreciated as the brain was traditionally considered an immune-privileged organ. Recently, this notion has shifted as increasing evidence demonstrates the capacity of the CNS to mount a considerable immunogenic response. However, GBM is characterized by severe immunosuppression.^10^ Indeed, the literature supports the development of a chronic inflammatory microenvironment as playing a substantial role in gliomagenesis, disease progression, and aggressive invasion of tumor cells.^11^ While microglia and infiltrating macrophages are the major immune cells present in GBM,^12^ lymphocytes and cells of myeloid lineage, including regulatory T cells (Tregs) and myeloid-derived suppressor cells (MDSCs), are present and help drive tumor-mediated immunosuppression.^10^ Additional immune-escape mechanisms include activation of indoleamine 2,3 dioxygenase, dysregulation of antigen presentation, and myeloid cell suppression driven by signal transducer and activator of transcription factor 3 (STAT3) have been described. Thus successful therapeutic activation of the immune system in GBM has been limited. However, connections between molecular profiling and immunophenotyping may lead to the development of new personalized therapeutic strategies.

### Epidemiology and pathological classification

Gliomas are the most commonly diagnosed group of primary brain neoplasms comprised of several phenotypically and molecularly distinct tumor types, which are staged according to WHO guidelines and increasing malignancy from grade I to grade IV.^9,13^ Approximately 55% of all gliomas are classified as grade IV GBM, and although relatively rare (2–3 cases per 100,000 adults in the US and Europe, annually),^14^ these tumors represent a persistent clinical challenge. GBM develops rapidly and spontaneously, with few known risk factors, little implication for familial heredity (<1%), and a 5-year survival rate of <5%.^15,16^ Though it occurs most frequently in Caucasian adults over the age of 50 years, GBM can also occur in infants, children, and young adults. However, given the distinct genetic background and etiology of these tumors, we do not cover them in this review.^17^

**Fig. 1:**
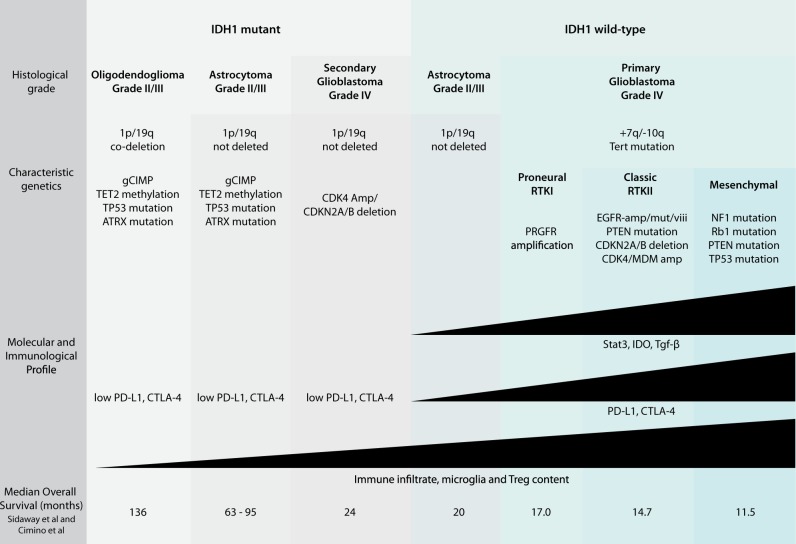
Molecular and immunological characterization of GBM^99–102^

Each glioma is characterized by a unique set of genetic and epigenetic changes that lead to upregulation or silencing of key biological pathways (Fig. 1). The downstream effects of these changes modulate complex signaling pathways and protein–protein interactions regulating tumorigenesis, proliferation, invasion, and apoptosis. Initial efforts to classify GBMs based on gene expression defined three major subtypes, referred to as proneural, proliferative, and mesenchymal.^8^ These subgroups were further refined through unsupervised clustering of transcriptome sequencing data. This approach revealed a hierarchical clustering of glioma samples, with the first major distinction between grade II and III oligodendrogliomas and grade IV GBMs, referred to as type O and type G, respectively.^18^ Type O tumors encompass the subgroup previously defined by Phillips as “proneural” and are enriched for gene expression patterns related to neurogenesis. Within the G type tumors, four further subclassifications, termed GA1, GA2, GB1, and GB2, were made. More recently, work from the The Cancer Genome Atlas (TCGA) Consortium separately described four molecular subtypes of grade IV GBM based on common mutations, deletions, amplifications, and methylation patterns, referred to as proneural (or RTKI), neural, classical (or RTKII), and mesenchymal.^7^ Across these classifications, differences in disease progression and survival are negligible, and only a handful of the identified mutations inform patient prognosis.

Mutation of *IDH1*, most frequently at exon 4 codon 132 (R132H), separates low-grade gliomas from high-grade GBM and is considered a strong prognostic marker.^19^ This gene encodes IDH1, an enzyme that catalyzes oxidative carboxylation of isocitrate to α-ketoglutarate and is a major cellular source of cytoplasmic NADPH. IDH1 mutation is most frequently detected in less aggressive low-grade gliomas and is associated with mutation of *TP53* and *ATRX*. In combination with the co-deletion of chromosomes 1p and 19q, *IDH* mutations are used to define oligodendrogliomas,^9^ although it is also found in 12% of all WHO grade IV GBMs, including 76% of secondary GBMs that progress from lower-grade tumors, and approximately 6% of primary adult GBMs, though typically in younger patients.^2,20,21^ As α-ketoglutarate is a co-factor for ten-eleven translocation (TET) histone demethylases, this mutation is associated with the CpG island methylator phenotype (g-CIMP).^22^ Interestingly, IDH1 wild-type low-grade gliomas often have compensatory methylation of the *TET2* promoter.^23^ In general, *IDH* mutations correlate to improved prognosis and increased overall survival, averaging 7 years in low-grade gliomas.^24^ In primary IDH1-mutant GBMs, the overall survival nears 46 months, compared to 13 months in IDH wild-type tumors.^19,25^

In addition to IDH1, the methylation status of the *MGMT* promoter is routinely assessed in glioma patients. MGMT is a DNA repair protein that contributes to therapeutic resistance, while silencing of this locus through hypermethylation of the promoter is predictive of an improved response to temozolomide (temodol) treatment. Although reports on the association between *MGMT* promoter methylation and progression-free survival are varied in their conclusions, meta-analysis of clinical data shows improved overall survival in methylated patients regardless of therapeutic intervention.^26,27^ These data suggest an additional functional significance for loss of MGMT expression, outside of its known role in chemo-sensitization. Interestingly, and in contrast to other molecular markers of GBM, longitudinal studies suggest that this epigenetic change is stably maintained throughout treatment and remains prognostic of treatment response even in recurrent tumors.^16^

Another prognostic marker associated with increased survival is mutation and loss of expression of the gene *ATRX*, which encodes a member of the SWI/SNF2 family of chromatin-remodeling proteins. Though rarely mutated in adult primary GBM, it is mutated at a higher frequency in pediatric and secondary GBMs and in low-grade astrocytomas (grade II and III).^20,28,29^ While not the focus of this review, it should be noted that pediatric and adult GBMs are distinct with regards to both molecular characterization and epidemiology. While many pediatric GBMs harbor *ATRX* mutations, tumorigenesis in these patients is thought to be driven by missense mutations at K27 and G34 in *H3F3A*, the gene encoding histone 3.3.^7,30–32^ Mutations at K27 are associated with additional mutations in *HIST1H3b*, *ACVR1*, and *TP53* as well as *ATRX*. Although many of the same mutations are found in G34-mutated tumors, these are also associated with DNA hypomethylation.

In adult GBM, several well-defined genetic changes are linked to poor prognosis. These include amplification (~40% of GBMs) and mutation (~25% of GBMs and 50% of EGFR-amplified GBMs) of the EGFR locus, which defines the “classical” GBM subtype and correlates with invasive and more aggressive disease progression.^33,34^ Deletion of the locus encoding *CDKN2A* and *CDNK2B* is also identified in classical-type primary GBMs, and this deletion can drive progression of low-grade gliomas to GBM.^5^ With similar downstream effects on the p53/RB pathway as *CDKN2A/B* deletion, co-amplification of *CDK4* and *MDM2* occurs in both IDH mutant and wild-type gliomas and is associated with significantly decreased overall survival.^35^ Within IDH1 wild-type GBMs, the mean survival for patients with CDK4/MDM2 amplification is reported to be 6.6 months, as compared to 12.7 months in non-amplified patients.^7,36–38^ In addition, mutation of the *TERT* promoter was recently identified as a marker of poor prognosis. Enriched in older patients and identified in approximately 40% of grade II/III gliomas^38^ and up to 75% of grade IV GBMs,^25^ this may represent a new hallmark of GBM. When coupled with *EGFR* mutations, *TERT* promoter mutations were associated with shorter overall survival. In contrast to patients with mutations in both *TERT* and *EGFR* genes, overall survival in *TERT/EGFR* wild-type patients (EGFR-non-amplified) was nearly twice as long (13 months vs 26 months).

Because screening for such molecular changes is now required in standard pathological analysis,^9^ improved detection methods are needed. Although clinical tools for array comparative genomic hybridization (aCGH), such as Onco-copy, are now more reliable even when using formalin-fixed paraffin-embedded sections, this technique cannot differentiate between low-level population changes and clonal amplification in small subpopulations.^39,40^ Recently, a combinatorial approach using aCGH and Onco-map, mass spectrometry-based mutation genotyping, demonstrated success in detecting mutations (IDH1, IDH2, TP53, phosphatase and tensin homolog (PTEN)), amplifications (*EGFR*, *PDGFRA*, *MET*), and deletions (*EGFRvIII*, *PTEN*, 1p/19q) at clinically relevant GBM loci.^40^ Interestingly, this method identified distinct profiles in GBM patients aged ≤40 years as compared to those aged ≥40 years.

### GBM heterogeneity—insights from NGS

Analysis of GBM through NGS reveals that the most commonly identified GBM mutations, amplifications, and deletions converge on three key signaling pathways, phosphoinositide-3 kinase/AKT/mammalian target of rapamycin (mTOR), Ras/RAF/mitogen-activated protein kinase, and p53/Rb.^4,5,7,32^ These include amplifications of genes encoding receptor tyrosine kinases (RTKs), such as *PDGFR* and *EGFR*, in proneural and classic-type GBMs, respectively, as well as mutation of *TP53*, *PTEN*, and *CDK4*. Logically, these data spurred drug development efforts focused on these pathways. Early compounds targeting mTOR seemed promising, though in vivo evaluation of first-generation mTORC1 inhibitors revealed only a transient response and no increase in progression-free or overall survival.^21,41–43^ Likewise, clinical trials of single agents targeting RTKs including EGFR and platelet-derived growth factor receptor (PDGFR) failed to improve prognosis.^41,44–46^ Indeed, evidence of clonal subpopulations harboring differential mutation and amplification of RTKs were identified through single-cell RNA-seq^40,47,48^ and constitutive pathway activation driven by *PTEN* deletion^49^ suggests that multi-pronged approaches and drugs targeting downstream factors may be more effective. Thus far, the complex molecular circuitry of GBM and the difficulties in deciphering datasets generated through high-throughput sequencing have precluded the development of patient-specific molecular profiles that inform targeted therapies for GBM.^50^

Though touted as a tool for increasing our understanding of GBM heterogeneity, single-cell RNA-seq data continue to add frightening levels of complexity to this disease. Such studies in low-grade glioma suggest that the majority of tumor cells follow the developmental hierarchy of glial differentiation programs with influence from the surrounding microenvironment. However, a small subpopulation of undifferentiated stem-like cells drive tumor growth and recurrence.^51^ Reflective of tumor evolution, an increase in the prevalence of both undifferentiated malignant cells and microglia is observed with increasing tumor grade.^37^ While cells in primary gliomas often share genetic signatures, longitudinal single-cell analysis suggests diversification increases with geographic location and reflects discrete mechanisms of therapeutic resistance.^45,52,53^ Tumors recurring adjacent to the primary lesion are more likely to possess the same genetic alterations, suggestive of intrinsic mechanisms of resistance. In contrast, distant recurrences are more often genetically distinct, likely due to acquired or treatment-induced resistance mechanisms. In addition, high-resolution sequencing of GBM methylation patterns, through bisulfite sequencing (BS-seq) and oxidative BS-seq in cells from discrete geographic regions revealed epigenetic spread, with genetically distinct cells from the tumor periphery carrying the same hypermethylation profiles as those from the tumor core.^54^ Thus, while therapeutics targeting truncal mutations (present in all cells) may be most effective in eliminating tumor mass, drugs targeting late-arising mutations or epigenetic changes may be necessary to combat the invasive spread of GBM.

In addition to the identification of molecular heterogeneity, NGS efforts improved characterization of the immunological profile of GBM, leading to expanded efforts in the development of immunotherapy. Interestingly, single-cell sequencing of tumor cells depleted of CD45+ inflammatory cells revealed four meta-signatures representing clustered pathways that vary synchronously across cells within individual tumors.^55^ One of these four signatures is characterized by enrichment for genes involved in complement system activation. Though unexpected in GBM cells, differential expression of the C3 complement gene was independently verified by further single-cell sequencing and other methods.^56,57^ Though the complement system is not extensively characterized in the context of GBM, expression of the C3 protein is thought to attract MDSC and promote immunosuppression.^58^ Further GBM single-cell profiling revealed transcriptionally distinct populations of myeloid cells at the infiltrating edge, compared to those within the tumor core.^59^ This finding, along with the surprising consistency in transcriptional signatures from infiltrating tumor cells across patients, suggests the existence of a common invasive mechanism that may involve immune cells.

The presence and diversity of tumor-infiltrating T cells can influence the balance between tumor-mediated immunosuppression and antitumor immune system activation (i.e., Tregs vs CD8+ T cells). The recent development of new bioinformatic approaches to quantify tumor-infiltrating T cell phenotypes from NGS data offers a means to understand tumor immunology on a patient-specific basis and may predict the response to immunotherapy.^60–62^ Likewise, NGS of T cell repertoires from tumor-infiltrating lymphocytes reveals different levels of T cell receptor (TCR) diversity and prevalence as compared to those present in the peripheral blood.^63^ In several cancers, T cell diversity has been shown to correlate with mutational burden in the tumor^6^; however, a recent study in GBM contradicts this finding.^64^ Specifically, this study reports the local environment as the major source of heterogeneity contributing to T cell diversity, and the authors suggest that tumor-specific neoantigens may not stimulate lymphocyte activation in GBM as they do in other tumor types.^64^ As such, it remains unclear whether the identification of cancer-specific antigens and tumor-reactive T cell clonotypes has therapeutic potential in glioma.

### Immunosuppressive profile of GBM

While cancers in other organs have benefited from immunotherapies, which can exploit either the innate or adaptive immune system, GBM has seen less progress to date. Characterization of GBM and its microenvironment suggest the preferential activation of tolerance pathways versus antitumor pathways, promoting tumor growth and invasion. Persistent secretion of immunosuppressive factors, including interleukin (IL)-1 and transforming growth factor (TGF)-β from the tumor, inhibits lymphocyte activity, and release of colony-stimulating factor-1 and IL-10 leads to activation and M2-type polarization of microglia.^65^ Additional secreted factors, such as vascular endothelial growth factor (VEGF; along with IL-10), nitric oxide, and prostaglandin E, can inhibit dendritic and natural killer (NK) cells, respectively. Through expression of these cytokines and additional immune-evasion strategies, GBM induces a state of systemic immunosuppression that promotes rapid tumor growth, invasion, and therapeutic resistance.

Interestingly, recent NGS data revealed heterogeneity in immune infiltrates,^60,61,63^ regional differences in macrophage activation,^56,66^ and utilization of different immunosuppressive mechanisms within the major GBM subtypes.^67^ In a detailed fluorescence-activated cell sorter-based study, Amankulor and colleagues demonstrated decreased CD45+ immune cell infiltration in human *IDH1* mutant tumors as compared to wild-type tumors.^68^ This included significant reductions in microglia, tumor-associated macrophages, T cells, B cells, and dendritic cells. Combined with TCGA data supporting decreased transcription of immune cell chemotaxis pathways, these data suggest that mutant IDH-driven changes in the tumor-associated immune cell component may contribute to the differential survival times observed in mutant and wild-type glioma patients. In similar studies, Kohanbash and co-workers demonstrated that *IDH* mutations reduced the levels of CXC motif chemokine ligand 10 and STAT1, and suppressed T cell accumulation in GBM tumors.^69^ These effects could be reversed by IDH-C35, an inhibitor of mutant IDH1, which enhanced the effectiveness of vaccine immunotherapy. Other studies demonstrate higher levels of programmed death ligand 1 (PD-L1) expression and enrichment of immune activators and suppressors, as well as increased CD3+/CD8+ T lymphocytes in mesenchymal as compared to proneural GBMs, suggesting that this subtype may be more immunoreactive by nature and therefore more amenable to immunotherapy.^70^

The role of immune checkpoint receptors programmed death 1 (PD-1), cytotoxic T lymphocyte antigen (CTLA-4), and T cell immunoglobulin mucin-3 (TIM-3) in GBM are of particular interest due to their expression patterns in GBM and the recent development of inhibitory therapeutics targeting PD-1 and CTLA-4. Activated CD4+ and CD8+ T cells are suppressed through engagement of the PD-1 receptor with its ligand PD-L1.^71–73^ PD-1 is also found on B cells, NK cells, and macrophages, and as such, constitutive PD-L1 expression on GBM tumor cells can induce widespread immunosuppression and alter the tumor microenvironment. Interestingly, data from GBM cell lines suggest that high levels of PD-L1 may correlate with mutations in PTEN.^52^ Unsurprisingly, given its widespread function, meta-analysis of GBM patients with high PD-L1 levels showed a correlation with decreased overall survival lengths, suggesting that this marker may have prognostic value.^74^ Recent data also suggest that extracellular vesicles that shed from GBM cells can bind PD-1, potentially independent of PD-L1 expression on their surface, to suppress T cell activation.^75^

GBM is also associated with lymphocytes and Tregs that constitutively express the surface receptor CTLA-4. Homologous to the co-stimulatory receptor CD28, CTLA-4 binds their shared ligands, CD80 or CD86, with increased affinity, preventing activation and proliferation of naive lymphocytes.^71,76^ Recent studies suggest that CTLA-4 expression may also hold prognostic value, though the methods used for CTLA-4 detection appeared to influence the statistical significance of these findings.^77^ Whether the mere presence of Tregs within GBM holds prognostic value is debated in the literature, with several studies presenting conflicting results.^78–80^ Data clearly demonstrate that increased proportions of Tregs among tumor-infiltrating T cells, which can be identified by the expression of FoxP3, are associated with increasing histological grade in gliomas and are particularly enriched within high-grade GBM. Within GBMs, high levels of FoxP3 are associated with poor prognosis, including decreased progression-free and overall survival.^78,79^

### New therapeutic avenues in GBM

**Fig. 2:**
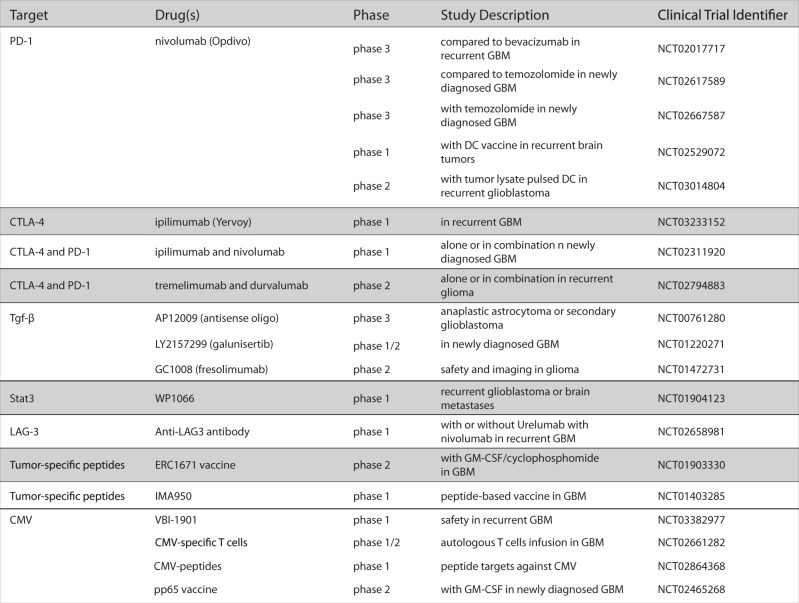
New trials in GBM (non-exhaustive list)

The continual evolution of GBM tumors, through genetic diversification and clonal expansion, results in the generation of cellular subpopulations with differential response to therapies and thus represents a major factor contributing to therapeutic resistance and failure of rationally targeted drugs (Fig. 2). Thus researchers and clinicians have faced repeated failures of promising drugs, including Rintega (rindopepimut), an EGFRviii vaccine, and Opdivo (nivolumab), a PD-1 inhibitor, which despite promising Phase-1 and -2 trials,^81,82^ recently missed Phase-3 endpoints and failed to significantly increase overall survival compared to controls. Recognition that GBM is continually changing recently led to longitudinal studies of responsive and non-responsive tumors, as well as the creation of the Glioma Longitudinal Analysis Consortium.^53,83^ These studies aim to better characterize the process of GBM recurrence in an effort to understand therapeutic resistance and identify new targets for intervention.

**Fig. 3:**
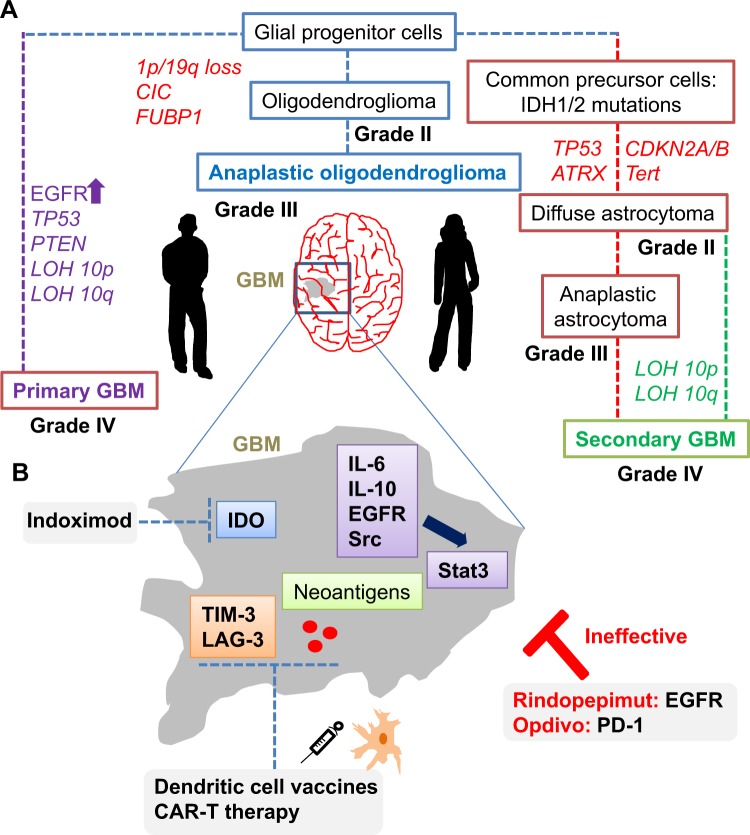
Immunogenetics of GBM and future emerging drug targets. **a** Quantitative analysis of copy number, mutational status, promoter methylation, and deletions are now established genes implicated in the pathophysiology of different GBM subtypes (including EGFR, ATRX, CDK4, CDKN2A/B, and Tert). In addition, longitudinal studies suggest that genetic diversification occurs as tumors evolve and recur. **b** Emerging NGS analyses has suggested novel signaling, inflammatory, and immune pathways that may vary within tumors of different molecular GBM subtypes. These data, combined with the success of immunotherapies in other cancer types, provide hope for new GBM-based immunovaccines and CAR-T therapies

Molecular hubs that connect aberrant signaling pathways with immunomodulation in GBM are considered promising targets. Stat3 is one such molecule, which is induced in response to cytokines such as IL-6 and IL-10, and activated downstream of tyrosine kinase receptors, such as EGFR and Src (Fig. 3a). In a feedback loop, activated Stat3 can transcriptionally regulate cytokine expression in immune cells, leading to constitutive Stat3 signaling.^84^ Global effects of Stat3 signaling on the immune system include the inhibition of dendritic cell maturation, downregulation of major histocompatibility complex class II molecules, and stimulation of Tregs.^84–86^

Indoleamine 2,3-dioxygenase (IDO), which plays an integrated role in tumor metabolism and immunity, is another promising target for new therapeutics. IDO is involved in tryptophan (Trp) metabolism that is expressed from the tumor itself, as well as from the stroma and antigen-presenting cells and macrophages, mediating antitumor immune responses.^6,87–89^ Through depletion of Trp and accumulation of the metabolic by-product kynurenine, high levels of IDO can lead to the inactivation of NK cells, suppression of tumor-specific T cells, and activation of Tregs (Fig. 3B). Preclinical models for studying tumor autoimmunity reveal new roles for IDO, which may dictate the balance between antitumor and tolerance in response to chemotherapy and cell death.^88^ In addition, IDO prevents the antitumor effects of T cell-dependent complement deposition, and inhibition of IDO was shown to reactive this process.^90–92^

Although high levels are IDO are associated with poor outcomes in a variety of cancers, trials of IDO inhibitors, such as indoximod alone, have shown limited success. However, in combination with temozolomide and other chemotherapies, such drugs may demonstrate increased efficacy.

Yet another molecular hub in GBM, Tgf-β is a cytokine expressed in numerous cell types with diverse biological functions ranging from proliferation to regulation of stemness and tumor suppression. Though highly expressed in glioma tissue and tumor-associated microglia, Tgf-β is not expressed in the normal brain.^93^ This expression pattern combined with studies showing that downstream Smad activation leads to PDGF-b-driven proliferative and invasion is suggestive of a role in tumor initiation.^94^ In addition, Tgf-β can upregulate VEGF, mediating angiogenesis, and can promote generation of Tregs. Within cytotoxic T lymphocytes, studies demonstrate direct binding of TGF-β to the promoter regions of genes regulating cytotoxicity, including interferon-g, Fas ligand, and granzyme B.^95^

Other therapeutic targets of interest include two additional immune checkpoint inhibitors, Tim-3 and lymphocyte-activation protein 3 (Lag-3). Similar to PD-1, Tim-3 is a marker of T cell exhaustion, and Lag-3 is a negative regulator of T cell and NK cell expansion.^96^ Both factors promote generation of Tregs.^71^ However, inhibitors of these factors have just been initiated in clinical trials.^97^ Tumor-specific dendritic cell vaccines and those targeting peptides overexpressed on GBM cells, such as cytomegalovirus, are in trials and chimeric TCR therapies are under development. While promising, active immunotherapy approaches in GBM must overcome the suppressive tumor microenvironment and mount a response great enough to induce lasting immunity without causing cerebral edema.^71,98^
